# Serum Neurofilament Light Chain and GFAP in Amyotrophic Lateral Sclerosis on a Commercial ECLIA Platform

**DOI:** 10.1002/mus.70397

**Published:** 2026-09-04

**Authors:** Nicholas S. Streicher

**Affiliations:** ^1^ Department of Neurology Georgetown University Washington DC USA

**Keywords:** amyotrophic lateral sclerosis, biomarkers, clinical practice, ECLIA, GFAP, implementation science, monitoring, neurofilament light chain, tofersen

## Abstract

**Introduction/Aims:**

In amyotrophic lateral sclerosis (ALS), SIMOA‐based studies show that baseline serum neurofilament light chain (NfL) predicts the revised ALS Functional Rating Scale (ALSFRS‐R) decline rate and survival. The Roche Elecsys electrochemiluminescence immunoassay (ECLIA) reports values approximately six‐fold lower; cross‐platform comparison confirms comparable performance, but serial clinical‐practice data remain limited. We assessed whether ECLIA NfL correlates with the ALSFRS‐R decline rate under the sampling conditions of clinical practice, and whether GFAP or S‐100B adds prognostic or disease‐specific information.

**Methods:**

We retrospectively analyzed 58 patients with medical‐record‐confirmed ALS at an academic center (2022–2026). Serum NfL, GFAP, and S‐100B were measured on the Roche Elecsys ECLIA. The NfL‐ALSFRS‐R correlation was assessed within matching windows; serial NfL was examined in patients with repeat draws.

**Results:**

First‐per‐patient NfL median was 7.06 pg/mL (IQR 4.06–17.30). NfL correlated strongly with the ALSFRS‐R decline rate (Spearman *r* = 0.704, *n* = 31; *r* = 0.809 within 90 days, *n* = 17; both *p* < 0.0001). Fast progressors had 3.7‐fold higher mean NfL than slow progressors (17.10 vs. 4.64 pg/mL). NfL showed no correlation with King's clinical stage (*r* = 0.00). GFAP correlated with age but not with progression rate or disease duration; S‐100B showed no association with progression.

**Discussion:**

Serum NfL on a commercial ECLIA platform retained its strong correlation with progression rate despite unstructured sampling, replicating SIMOA‐based findings at platform‐specific values. NfL tracked the rate of decline rather than the accumulated disease state; GFAP and S‐100B added no prognostic or disease‐specific information.

## 1 Introduction

Neurofilament light chain (NfL), a 68‐kDa neuronal cytoskeletal protein released into blood after axonal damage [1, 2], is extensively validated as a prognostic biomarker for amyotrophic lateral sclerosis (ALS) [3, 4, 5, 6, 7, 8] and recognized by the US Food and Drug Administration (FDA) as a surrogate endpoint [9, 10, 11, 12]. The initial SIMOA‐based blood and cerebrospinal fluid NfL studies discriminated ALS from mimics and healthy controls, with higher baseline values predicting faster functional decline and shorter survival [2, 3, 4, 6, 7, 8, 13, 14, 15, 16, 17, 18]. The presymptomatic carrier studies of Benatar and colleagues showed that serum NfL rises months before the first clinical weakness and plateaus around diagnosis, anchoring its interpretation to the disease trajectory [19, 20]. That evidence comes from research conditions: selected patients, protocol‐mandated assessments, and complete data capture [5].

Performance, however, must also be established on the commercially available assay used in clinical care, as normative values and diagnostic thresholds are platform‐specific. The Roche Elecsys electrochemiluminescence immunoassay (ECLIA) yields values approximately six‐fold lower than SIMOA; Mondesert and colleagues subsequently confirmed strong cross‐platform agreement and comparable diagnostic and prognostic performance in a four‐platform comparison, including for the ECLIA assay [21], with consistent between‐assay differences in head‐to‐head comparison [22], although ECLIA data in ALS in the clinical setting remain limited.

We therefore analyzed ECLIA‐based serum NfL as the primary marker, with GFAP and S‐100B, in patients with ALS under routine clinical care. The primary objective was to test whether ECLIA NfL correlates with the revised ALS Functional Rating Scale (ALSFRS‐R) decline rate; secondary objectives were to define its platform‐specific distribution and to evaluate whether GFAP or S‐100B adds prognostic or disease‐specific information. We hypothesized that the rate relationship established under research conditions would persist in unstructured clinical sampling.

## 2 Methods

### 2.1 Study Design

We conducted a retrospective analysis of electronic health record (EHR) data from a single tertiary academic center. The IRB‐approved protocol included a waiver of informed consent for retrospective EHR review.

### 2.2 Inclusion and Exclusion Criteria

Candidate patients were identified by ICD‐10 coding (G12.20, G12.21, and G12.29) combined with serum biomarker test ordering at a tertiary ALS referral center between January 2022 and March 2026. Each candidate underwent individual medical‐record review to confirm an ALS diagnosis. Patients whose records did not support ALS were reassigned to their actual diagnosis and excluded.

### 2.3 Sampling Procedures

Patients were sampled from routine clinical practice. Biomarkers were ordered at the discretion of the treating clinician, so per‐modality availability varied across the cohort.

### 2.4 Sample Size, Power, and Precision

No a priori power calculation was performed. The sample size was fixed by the number of eligible patients in the study period, all of whom were included.

### 2.5 Measures and Covariates

All biomarker measurements were performed by LabCorp (Burlington, NC) using the Roche Elecsys ECLIA platform. NfL was assayed via test code 505115 (analytical sensitivity 0.241 pg/mL, inter‐assay CV < 5%); GFAP and S‐100B were measured on the same platform. Where the laboratory information system recorded multiple entries for a single draw on the same calendar date, these were treated as one timepoint; only measurements on different calendar dates were considered repeat draws for longitudinal analysis. Laboratory dates reflect result dates.

Item‐level ALSFRS‐R scores were extracted from structured clinical‐note templates. For patients with assessments on two or more dates, the rate of decline was calculated as (first ALSFRS‐*R* − last ALSFRS‐R) divided by months between them, in points lost per month, over each patient's full assessment interval. Patients were classified as fast progressors (> 1 point/month decline), intermediate (0.5–1 point/month), or slow (< 0.5 point/month) [23]. One patient with a single structured assessment and documented interval functional decline in the clinical notes was classified as a fast progressor by clinician adjudication. King's clinical stage was derived from item‐level ALSFRS‐R scores (bulbar, Items 1–3; upper limb, Items 4–5; lower limb, walking and stair‐climbing items; Stage 4, respiratory‐insufficiency item below 4), without separation of Substages 4a and 4b.

Treatment and genetic information were ascertained from available clinical notes. Age and time from symptom onset were recorded as covariates for the GFAP analysis.

### 2.6 Statistical Analysis

Spearman rank correlation assessed the association between first‐per‐patient NfL and the ALSFRS‐R decline rate, with a sensitivity analysis restricting NfL to within 90 days of the rate‐defining assessments (the rate itself was calculated over each patient's full assessment interval), and between first‐per‐patient NfL and the nearest contemporaneous ALSFRS‐R score (within 90 days). Correlation strength was interpreted per Schober et al.: 0.10–0.39 weak, 0.40–0.69 moderate, 0.70–0.89 strong [24]. Between‐group comparisons used the Mann–Whitney U test. Within‐subject NfL trajectories were summarized descriptively. Within‐patient GFAP slopes were estimated for patients with two or more draws using a within‐patient (fixed‐effects) estimator pooled across patients. SIMOA‐equivalent values were estimated using the published Elecsys‐to‐SIMOA regression of Booth et al. (SIMOA = 6.566 × Elecsys −0.812; *r* = 0.991) [25], consistent with the approximately six‐fold difference reported by Mondesert et al. [21]. *p* < 0.05 was considered significant.

Serum GFAP was examined against age, the ALSFRS‐R decline rate, cumulative functional loss (48 minus the contemporaneous ALSFRS‐R score), and time from symptom onset, using first‐per‐patient values. To separate disease‐related change from normal aging, GFAP was age‐adjusted to a reference age of 60 years using a published healthy‐control rate of ~1.5% per year. The fraction of GFAP values within the non‐ALS comparator range was determined against the 90th percentile of the peripheral‐neuropathy and unclassified cohorts.

## 3 Results

### 3.1 Cohort Characteristics

The final cohort comprised 58 patients with medical‐record‐confirmed ALS; demographic and clinical characteristics are summarized in Table 1. The cohort includes one presymptomatic pathogenic‐variant carrier under clinical surveillance. Among the 50 patients with available clinical notes, disease‐modifying treatment was documented as riluzole in 9, edaravone in 10, and sodium phenylbutyrate‐taurursodiol in 2; one patient with SOD1‐associated ALS was receiving tofersen.

**TABLE 1:** ALS cohort demographics (*n* = 58).

Characteristic	Value
Age, years: mean ± SD; median (range)	63.7 ± 14.4; 64 (27–92)
Sex, *n* (%): male/female/unknown	30 (52)/23 (40)/5 (9)
Race (*n* = 53), *n* (%)	
White	22 (42)
African American	17 (32)
Asian	2 (4)
Other	3 (6)
Declined or unknown	9 (17)
Ethnicity (*n* = 53), *n* (%)	
Non‐Hispanic	35 (66)
Hispanic	0 (0)
Declined or unknown	18 (34)
King's clinical stage at first assessment (*n* = 43), *n* (%)	
Stage 1	9 (21)
Stage 2	14 (33)
Stage 3	18 (42)
Stage 4	2 (5)
ALSFRS‐R at first assessment (*n* = 43): median (range)	36 (13–48)
ALSFRS‐R decline rate, points/month (*n* = 32): median (IQR)	0.20 (0.08–0.97)

Available measurements differed by patient and analysis. Fifty‐seven patients had at least one NfL value (80 total), 29 had at least one GFAP value (39 total), and 14 had at least one S‐100B value (18 total). Forty‐two patients had at least one ALSFRS‐R assessment (190 total), of whom 32 had a calculable decline rate over a median 19 months of assessment follow‐up; 31 had both an NfL value and a decline rate, and 13 had NfL on two or more distinct dates; the median interval between the first NfL value and the nearest ALSFRS‐R assessment was 8 days (IQR 2–44), with 27 of 31 within 90 days.

### 3.2 Serum NfL Distribution

First‐per‐patient serum NfL spanned a 65‐fold range with high dispersion (Table 2). Applying the published ECLIA‐to‐SIMOA conversion [25] places the median at approximately 46 pg/mL (IQR 26–113).

**TABLE 2:** Serum NfL distribution: First‐per‐patient (*n* = 57).

Statistic	ECLIA	SIMOA‐equivalent*
Median (pg/mL)	7.06	~46
IQR (pg/mL)	4.06–17.30	~26–113
Min–Max (pg/mL)	0.96–62.20	~5–408
Mean (pg/mL)	11.1	~72
CV	99%	—

* SIMOA‐equivalent via Booth et al. regression (SIMOA = 6.566 × Elecsys −0.812; *r* = 0.991) [25].

### 3.3 NfL And ALSFRS‐R Progression Rate

Baseline NfL correlated strongly with the ALSFRS‐R decline rate, and the association was essentially unchanged when restricted to NfL drawn within 90 days of the rate‐defining assessments (Table 3; Figure 1). Fast progressors had a 3.7‐fold higher mean NfL than slow progressors (17.10 vs. 4.64 pg/mL). NfL showed no significant correlation with the contemporaneous ALSFRS‐R score, age, or item‐derived King's clinical stage, did not differ by race (*p* = 0.94), and the decline‐rate association was unchanged after adjustment for age and sex (Table 3).

**TABLE 3:** Correlations of serum biomarkers with clinical measures.

Comparison	*n*	Spearman *r*	*p*	Strength
NfL vs. ALSFRS‐R decline rate	31	0.704	< 0.0001	Strong
NfL vs. decline rate, within 90 days	17	0.809	< 0.0001	Strong
NfL vs. decline rate, age‐ and sex‐adjusted	31	0.73	< 0.0001	Strong
NfL vs. contemporaneous ALSFRS‐R score	34	−0.27	0.13	Not significant
NfL vs. age	57	0.23	0.10	Not significant
NfL vs. King's clinical stage	32	0.00	0.99	Not significant
GFAP vs. age	28	0.76	< 0.001	Strong
GFAP vs. ALSFRS‐R decline rate	15	0.13	0.65	Not significant
GFAP vs. cumulative functional loss	20	0.12	0.61	Not significant
GFAP vs. time from symptom onset	13	0.09	0.76	Not significant

*Note:* Spearman rank; strength interpreted per Schober et al. [24].

**FIGURE 1:**
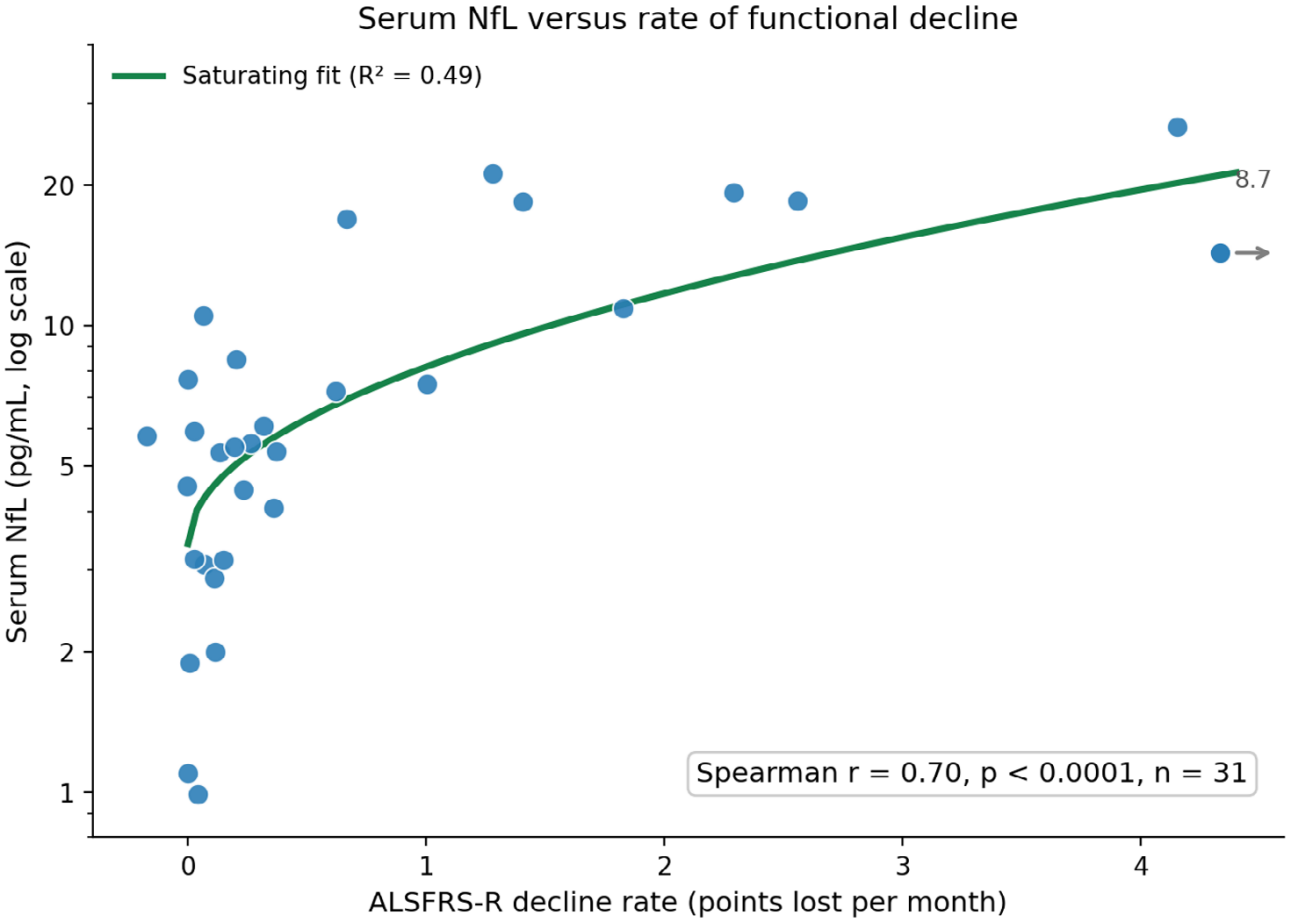
First‐per‐patient serum NfL versus the ALSFRS‐R decline rate (*n* = 31); statistics, fitted curve, the off‐scale point, and progressor definitions are given in the Results.

### 3.4 Longitudinal NfL

Thirteen patients had serial NfL on two or more distinct dates, including one presymptomatic gene carrier under clinical surveillance; individual trajectories are shown with group means for fast and slow progressors in Figure 2. One patient was classified as a fast progressor by clinician adjudication of documented interval decline; one patient lacked ALSFRS‐R data.

**FIGURE 2:**
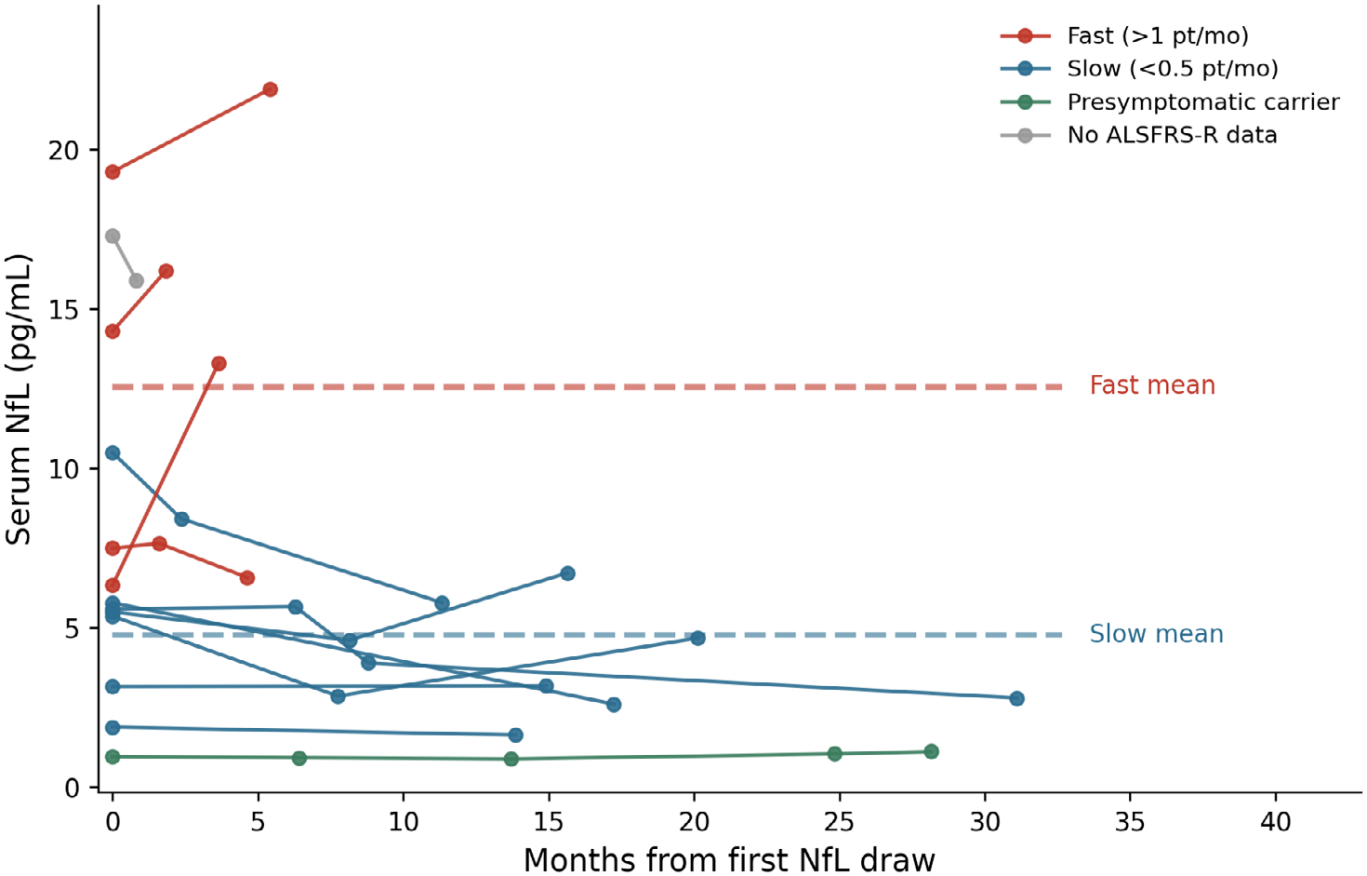
Longitudinal serum NfL. Serial values in the 13 patients with two or more draws.

### 3.5 GFAP And S‐100B

GFAP was available in 29 patients (39 values; median 71.70 pg/mL) and S‐100B in 14 (18 values; median 53.05 pg/mL). Most GFAP values were not elevated: 24 of 29 (83%) fell within the range of the non‐ALS neurologic comparators. GFAP correlated strongly with age but not with the ALSFRS‐R decline rate, cumulative functional loss, or time from symptom onset (Table 3; Figure 3).

**FIGURE 3:**
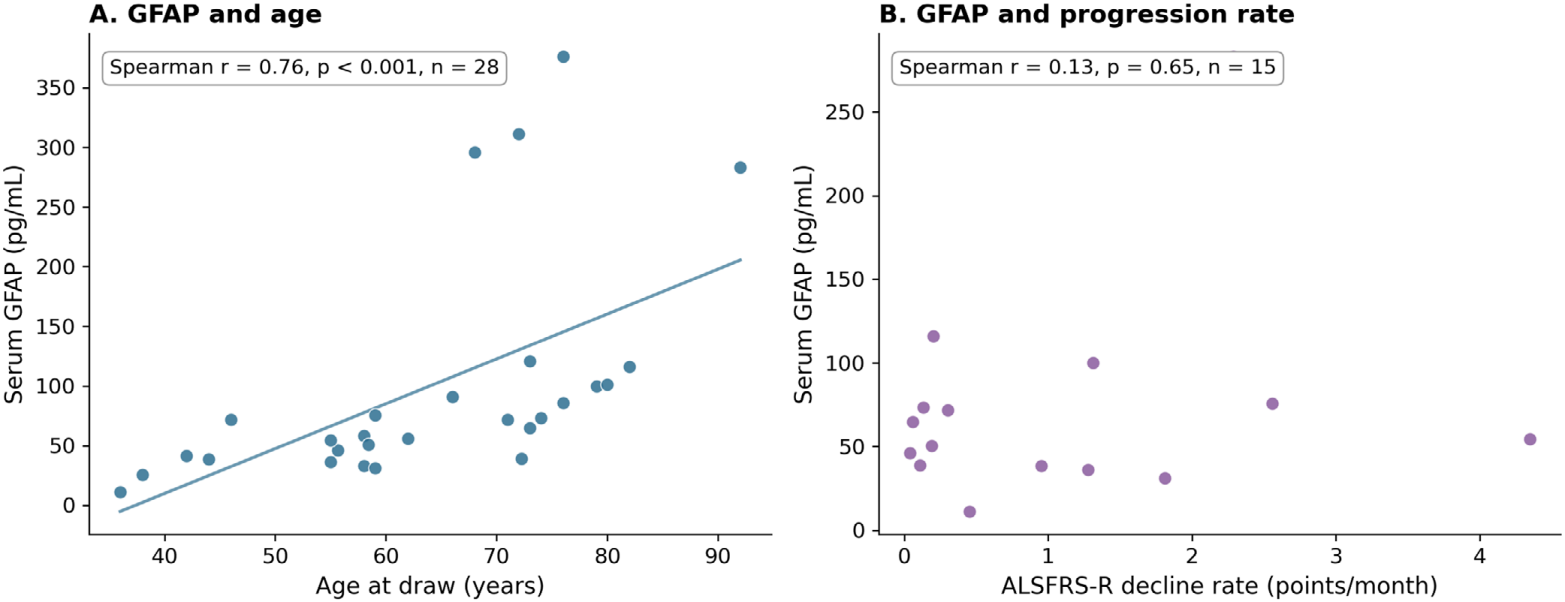
Serum GFAP, age, and progression rate. (A) First‐per‐patient GFAP versus age at draw (Spearman *r* = 0.76, *p* < 0.001, *n* = 28). (B) First‐per‐patient GFAP versus the ALSFRS‐R decline rate (*r* = 0.13, *p* = 0.65, *n* = 15).

Among patients with two or more GFAP draws, values rose or were stable in most and fell in none, but the within‐patient change was small and not significant (pooled slope *p* = 0.11); age‐normalization left no association with cumulative functional loss or disease time. S‐100B showed no association with the decline rate; an inverse association with age in this small subset is of uncertain significance (Figure S1).

## 4 Discussion

Serum NfL measured on the commercial ECLIA platform maintained its prognostic value in clinical practice. Baseline values correlated strongly with the ALSFRS‐R decline rate but showed no correlation with the absolute ALSFRS‐R score or the item‐derived King's clinical stage. GFAP scaled with age rather than the motor disease, and S‐100B showed no association with progression in a preliminary subset.

### 4.1 Comparison With Previous Literature

Mondesert and colleagues showed comparable diagnostic and prognostic performance for ECLIA NfL using single baseline values in an enrolled cohort [21], with consistent between‐assay differences in head‐to‐head comparisons [22]. In their study, GFAP added no diagnostic or prognostic value as a single marker, matching our findings [21]. The present data add sampling outside any research protocol, correlation with the ALSFRS‐R decline rate, and serial measurement, which their discussion identifies as a necessary step before routine use.

Benatar and colleagues, following gene carriers from before symptom onset, defined the NfL trajectory: Rising before the first weakness, plateauing by diagnosis, and holding stable at a level set by the rate of ongoing injury [19, 20, 26]. The plateau reflects an equilibrium between release from degenerating axons and blood clearance [19]. In those cohorts, the steady‐state level tracked the future rate of decline rather than the contemporaneous score [5, 19], the pattern reproduced here on a commercial platform. As NfL correlates with the extent of both upper and lower motor neuron involvement [27], it is lower in primary lateral sclerosis, where degeneration is restricted to corticospinal pathways [28]; whether a central source also contributes remains unresolved [29, 30, 31, 32]. Our serial data extend the model into a later phase those cohorts rarely sampled: the substantial declines occurred in established or advanced disease, none in a patient receiving tofersen, consistent with late loss of the motor axons that release the marker [33, 34, 35]. In a deteriorating patient, a falling value may represent depletion rather than improvement.

The SIMOA and Roche assays are comparably sensitive but differ in calibration and dynamic range: ECLIA values run approximately six‐fold lower, and an elevation notable on one scale may appear modest on the other [21, 22]. Published regression converts values between platforms [25], but prognostic cutoffs were validated per assay [5, 36], so interpretation anchors to the reporting platform's own reference intervals and cutoffs [21, 25]. In previous studies, validation cohorts enrolled near diagnosis, at the trajectory plateau, whereas clinical draws may occur at varied and unplanned points across the disease course. Many values fell within the reference range here, as in prior clinical cohorts [37], even in a disease with some of the highest average NfL values reported across platforms [2].

The GFAP findings are consistent with prior data across platforms. Neither progression rate, cumulative functional loss, nor disease duration correlated with GFAP; age did, and most values were normal, matching the age‐driven pattern reported on SIMOA and LUMIPULSE and the amyloid co‐pathology that raises GFAP in older adults [29, 38, 39]. GFAP is astrocyte‐derived and mainly reflects central nervous system damage [40]. It rises within hours of acute injury [41] and persists where astrogliosis is sustained, as in central neurodegenerative disease [40]. In ALS, the astroglial response is regionally restricted to motor pathways rather than diffuse, so the glial signal may not rise above the age‐related background.

### 4.2 Clinical Implications

Serum NfL changes across the ALS course: low before onset, climbing as axons degenerate, plateauing in established disease, and, in some patients here, falling late in the course. The value can be used diagnostically in early disease, where a high value supports active degeneration but a normal one cannot exclude ALS [37]; presymptomatically in at‐risk carriers, where serial values can detect rising pathology before phenoconversion [20, 42]; and prognostically in established disease, where it tracks the injury rate. The same value can therefore be useful at several stages of disease, but using it properly requires understanding its kinetics and the clinical context.

For treatment response, monitoring is established for tofersen in SOD1‐ALS [9, 10, 11]; thresholds for broader practice remain undefined, reflecting both limited treatment options and the absence of prespecified sampling timelines outside trials. Although a rising serial value signals an accelerating rate [19, 43], a single low value after symptom onset argues against a rapidly progressive process: it is equally compatible with indolent or late‐stage motor neuron disease, primary muscle disease, or an inflammatory neuropathy, which can run normal‐to‐high [44]. Prospective, protocol‐driven serial sampling in the clinical setting is needed to validate the trajectory as a monitoring endpoint.

### 4.3 Limitations

This single‐center retrospective study relied on clinical notes, which varied in completeness and quality and may not fully capture the clinical picture. Subset sizes varied by analysis according to data availability, limiting the precision of the longitudinal and multi‐biomarker estimates: Fewer than a third of patients had a repeat NfL draw, and the within‐patient GFAP analysis rests on six patients. Few patients declined at intermediate or rapid rates, so the correlation is anchored mainly by slow progressors and is estimated less precisely at the fast end of the range. Patients reach this clinic by referral, usually after diagnosis, which likely underrepresents early and rapidly progressive disease. The association is likely to generalize, but the distribution of progression rates will vary with site, clinic, and referral pattern. We did not measure amyloid, phosphorylated tau, or TDP‐43 species, so the anatomical source of serum GFAP cannot be localized. S‐100B data were sparse, showed no association with progression, and are confounded by non‐neural sources. Renal function and body mass also influence NfL, but documented renal disease was rare and body mass largely normal.

## 5 Conclusions

On a commercial ECLIA platform, serum NfL retained a strong correlation with ALS progression rate under the sampling conditions of clinical practice; GFAP and S‐100B added no prognostic or disease‐specific information. A single NfL value reports the rate of axonal injury, not its accumulated extent, and is interpretable only against assay‐specific reference intervals and clinical context. Further work should standardize calibration across platforms and define the optimal timing of NfL testing in the clinical setting.

## Author Contributions

**Nicholas S. Streicher:** conceptualization, investigation, writing – original draft, methodology, validation, visualization, writing – review and editing, formal analysis, project administration, data curation, resources.

## Funding

The author has nothing to report.

## Ethics Statement

This retrospective study was approved by the Georgetown‐MedStar Institutional Review Board (protocol no. STUDY00008842; initially approved October 10, 2025), with a waiver of informed consent for review of electronic health record data.

## Conflicts of Interest

N.S. has served as an internal speaker for Biogen, received research funding from Alnylam, and served as an internal speaker for AstraZeneca, all outside the submitted work.

## Supporting information


**Figure S1:** Serum S‐100B. (A) First‐per‐patient serum S‐100B versus age at draw (Spearman *r* = −0.66, *p* = 0.01, *n* = 13). (B) First‐per‐patient S‐100B versus the ALSFRS‐R decline rate (*r* = 0.10, *p* = 0.87, *n* = 5).

## Data Availability

De‐identified data supporting the findings are available from the corresponding author on reasonable request, subject to institutional data‐sharing agreements.
