# Supplementary material for: Serum Neurofilament Light Chain and GFAP in Amyotrophic Lateral Sclerosis on a Commercial ECLIA Platform
Source: Muscle Nerve. 2026 Sep 4;74(5):1398–405. doi: 10.1002/mus.70397 (PMC13644906; doi:10.1002/mus.70397)
Supplement: Supplementary file 1 [file MUS-74-1398-s001.docx]

# Supplementary Material

**Serum Neurofilament Light Chain and GFAP in Amyotrophic Lateral Sclerosis on a Commercial ECLIA Platform**

Nicholas S. Streicher, MD, MPH

**Supplementary Figure S1. Serum S-100B.** (A) First-per-patient serum S-100B versus age at draw (Spearman r = -0.66, p = 0.01, n = 13). (B) First-per-patient S-100B versus the ALSFRS-R decline rate (r = 0.10, p = 0.87, n = 5).
